# Stability and virucidal efficacies using powder and liquid forms of fresh charcoal ash and slaked lime against Newcastle disease virus and Avian influenza virus

**DOI:** 10.14202/vetworld.2019.1-6

**Published:** 2019-01-02

**Authors:** Sakchai Ruenphet, Darsaniya Punyadarsaniya, Tippawan Jantafong, Kazuaki Takehara

**Affiliations:** 1 Department of Immunology and Virology, Faculty of Veterinary Medicine, Mahanakorn University of Technology, Thailand; 2 Department of Veterinary Medicine, Laboratory of Animal Health, Faculty of Agriculture, Tokyo University of Agriculture and Technology, Japan

**Keywords:** alkaline agent, fresh charcoal ash, slaked lime, virucidal activity

## Abstract

**Aim:**

The present study was examined the virucidal activity comparison between fresh charcoal ash (FCA) and slaked lime (SL) against avian influenza virus (AIV) and Newcastle disease virus (NDV), using powder and liquid forms, either in the absence or presence of organic materials. In addition, both FCA and SL were evaluated for the persistence of virucidal activity in wet and dry conditions and stability of the solution.

**Materials and Methods:**

Two hundred milligrams of FCA or SL powders were mixed with 100 µl of AIV or NDV in the absence of organic material or 33% of organic materials. In the same time, 400 µl of 1%, 5%, or 10% solution samples were mixed with 100 µl of each virus and then incubated at room temperature for an indicated time. After that, the mixed solution was stop activity of sample using 500 µl of 1M Tris-HCl pH 7.2. Each treatment was titrated onto Madin-Darby canine kidney cells or chicken embryo fibroblasts for AIV or NDV, respectively, for determining the efficacy of viral inactivation. In addition, the stability of powder under the wet-dry condition and solution stability under room temperature was examined.

**Results:**

The results demonstrated that the FCA and SL in powder form could inactivate AIV and NDV even in the absence or presence of organic materials. In the liquid form, 5% and 10% of FCA could inactivate AIV and NDV either in the absence or presence of organic materials. Alongside, 1%, 5%, and 10% of SL could inactivate both viruses. 10% of FCA solution could inactivate virus at a shortest time when compared with other concentrations. In addition, the efficacy of wet-dry conditions of FCA was limited when compared with SL. On the other hand, it is demonstrated that the FCA solution was more stable and kept at room temperature longer than SL.

**Conclusion:**

The FCA may, hence, be used as an alternative virucide, while applying it to prevent spreading of poultry disease on commercial chicken farms and also backyard chickens, especially in developing countries, including in rural areas of Thailand.

## Introduction

Several viral diseases, especially avian influenza (AI) and Newcastle disease, have a strong negative impact on commercial chicken. Normally, the related viruses are shed from the respiratory and gastrointestinal systems of clinically or sub-clinically infected birds and circulate in the environment. Biosecurity on farms, such as cleaning and disinfection, is one of the best instruments to reduce the microbial load generally and the level of pathogens in particular, in poultry farms [1,2], especially the mentioned viruses. In general, several organic solvents, detergents, and disinfectants are applied for microorganism inactivation; however, their efficacy is decreased when contaminated with organic materials [2]. There were several trials to outline alternative materials for biosecurity enhancement, which are not affected by organic materials contamination. Several researchers adapted the alternative materials for biosecurity enhancement in spite of the presence of organic material contamination, using alkaline agents such as slaked lime (SL), bioceramic powder [3], scallop shell powder [3], calcinated eggshell [4], and nano-sized scallop shell powder [2].

Charcoal ash or wood ashes are waste products from restaurants and household after cooking, which have alkaline compounds such as calcite (calcium carbonate [CaCO_3_]), lime (CaO), and portlandite calcium hydroxide (Ca[OH]_2_) [5,6]. The alkalinity of charcoal ash is high, with pH from 9.3 to 13.5 [5].

The aims of the present study were to evaluate virucidal efficacies of fresh charcoal ash (FCA) against various chicken pathogens such as AIV and Newcastle disease virus (NDV), either in the absence or presence of organic materials, to evaluate their stability under wet and dry conditions, and to appraise solution stability for biosecurity application in chicken farms.

## Materials and Methods

### Ethical approval

Ethical approval is not applicable to this study.

### Sample preparation

The FCA powders prepared by burning wood charcoal and SL (Zapco^®^, Homeinter supply Co., Ltd., Nonthaburi, Thailand) were used for the present study. Both solution samples were prepared as 1%, 5%, and 10% dilutions using distilled water. A quantity of 1, 5, or 10 g of each powder was added to 100 ml of dW_2_ and centrifuged at 1750× g for 10 min. The resulting supernatants were used as 1%, 5%, or 10% solutions as described [4].

### Viruses and cells

Low pathogenic AI virus, namely A/duck/Aomori/395/04 H7N1 [7], and virulent vNDV, namely NDV/chicken/Asean Country/2013 [8], were propagated in chicken embryonic eggs. After allantoic fluid harvesting, stock viruses were aliquoted and kept at −80°C for testing. Madin-Darby canine kidney (MDCK) cells and chicken embryo fibroblasts (CEF) were used for AIV and NDV titration, respectively.

### Powder reaction

To determine virus inactivation, 200 mg of FCA or SL powders were mixed with 100 µl of AIV or NDV in the absence of organic material. In addition, for evaluating the presence of organic materials, 100 µl of each virus was mixed with 50 µl of fetal bovine serum (FBS), and then the mixture was added to 300 mg of each powder sample. After 3 min incubation at room temperature, the viruses were recovered with 900 µl or 850 µl of PBS, respectively, then centrifuged at 17,400× g for 3 min, and titrated onto MDCK cells or CEF for virus recovering [4].

### Liquid reaction

About 400 mL of 1%, 5%, or 10% solution samples were mixed with 100 µl of each virus and then incubated at room temperature for an indicated time such as 5 s, 30 s, 1 min, 3 min, 5 min, 10 min, 30 min, 1 h, or 2 h. After that, the mixed solution pH was neutralized with 500 µl of 1M Tris-HCl pH 7.2. Each sample treatment was titrated onto MDCK cells or CEF for AIV or NDV, respectively. 500 µl of FBS was added to 10 ml of each sample concentration as 5% organic materials representation. To confirm the neutralizing efficacy of Tris-HCl, it was added to each solution sample before virus adding, namely at 0 s. Each treatment was tested in triplicates, and the titers were shown in mean with standard error (mean ± SE).

### Virus titration

Each treated virus was diluted in 10-fold serial dilution using Eagle’s minimum essential medium (MEM, Nissui Pharmaceutical Co., Ltd., Tokyo, Japan) and inoculated onto MDCK or CEF cells containing equal volume of MEM with trypsin (Trypsin, Sigma, St. Louis, MO, U.S.A.) reaching final concentrations of 0.5 and 0 µg/ml, respectively. All of the inoculated cell plates were incubated at 37°C in 5% CO_2_ incubator and observed for the cytopathic effect twice a day for 3 days. At the end of the incubation period, the hemagglutinin activity of the culture supernatant was tested using 0.5% chicken red blood cells. The 50% tissue culture infectious dose (TCID_50_/ml) was determined by Behrens and Kärber’s method [2].

### Stability

The virucidal activity of FCA and SL stored under harsh conditions using wet-dry environmental transitions was also evaluated. A quantity of 3 g of FCA or SL powder in a 90-mm Petri dish was used for making suspensions in 10 mL with tap water, and the dish was kept at 37°C incubator for complete drying. The dried sample was collected and tested by powder reaction method. Resuspension and drying were repeated until virucidal efficacy ceased.

The stability of the solution sample was evaluated at 2, 4, 6, and 8 weeks post preparing and keeping at room temperature. Virus inactivation was determined within a 3-min incubation period.

### Inactivation analysis

The reduction factor (RF) was used for determining virus inactivation. The RF is calculated using the following equation: RF = t_pc_−t_a_; where t_pc_ is the titer converted into an index in log_10_ of the positive control, and t_a_ is the converted titer an index in log_10_ of the recovered virus from the treated sample. Inactivation of the virus was considered effective when RF was ≥3 log_10_ [2,9,10].

### Statistical analysis

The SE is the standard deviation of virus titer distribution. SE was calculated by standard deviation using Microsoft Excel.

## Results

The pH of 1%, 5%, and 10% FCA solutions was recorded by pH paper strip as 12.0, 12.5, and 13.0, respectively, while the overall concentration of SL was measured to be 12.5.

Table-1 shows that either FCA or SL powders could inactivate AIV and NDV both in the absence and presence of organic materials and reduced the virus titer to below the detection limit (2.5 log_10_ TCID_50_/ml).

**Table-1:** The results are shown as Log_10_ TCID_50_/ml (mean±SE) of avian influenza virus inactivating activity by means of reaction in the powder of fresh charcoal ash and slaked lime in the absence and presence organic materials.

Conditions	Newcastle disease virus				Avian influenza virus			
	
	Fresh charcoal ash		Slaked lime		Fresh charcoal ash		Slaked lime	
			
	Absence^a^	Presence^b^	Absence	Presence	Absence	Presence	Absence	Presence
t_pc_^c^	8.00±0.43	8.50±0.43	7.75±0.25	8.42±0.38	6.75±0.25	6.42±0.38	7.00±0.25	6.67±0.29
t_a_^d^	<2.50±0.00	<2.50±0.00	<2.50±0.00	<2.50±0.00	<2.50±0.00	<2.50±0.00	<2.50±0.00	<2.50±0.00
RF^e^	>5.50±0.43*	6.00±0.43*	>5.25±0.25*	>5.29±0.38*	>4.25±0.25*	>3.92±0.38*	>4.50±0.25*	>4.17±0.29*

a Absence of organic material.

b Presence of organic material as 33%.

c The titer converted into an index in log_10_ of treatment.

d The titer converted into an index in log_10_ of the recovered AIV from the treated tube.

e The reduction factor=t_pc_-t_a_.

* Inactivation regarded effective when RF was ≥3

The AIV inactivation is shown in Table-2. At 5% and 10%, FCA without organic material contamination could inactivate AIV within 30 min and 30 s, respectively. Even in the presence of organic materials, inactivation occurred within 2 h and 1 min, respectively. However, all concentrations of SL could inactivate AIV in the absence and presence of organic materials within 10 min and 30 min, respectively (Table-2).

**Table-2:** The results are shown as log_10_ TCID_50_/ml (mean±SE) of Avian influenza virus inactivating activity by means of reaction in 1%, 5%, and 10% fresh charcoal ash and slaked lime solution in the absence or presence organic materials.

Conditions	Fresh charcoal ash						Slaked lime					
	
	Absence organic materials			Presence organic materials^a^			Absence organic materials			Presence organic materials		
			
	1%^b^	5%	10%	1%	5%	10%	1%	5%	10%	1%	5%	10%
t_pc_^c^	7.25±0.50	7.17±0.58	7.42±0.88	7.42±0.29	7.17±0.58	7.42±0.88	7.92±0.52	7.58±0.52	7.58±0.52	7.92±0.52	7.58±0.52	7.58±0.52
0 s^d^	7.00±0.25	6.75±0.43	7.50±0.90	7.42±0.58	7.17±0.38	7.33±0.72	7.58±0.63	7.17±0.38	7.33±0.72	7.75±0.75	7.42±0.38	7.58±0.63
5 s^e^	NT^f^	NT	6.33±0.72	NT	NT	6.25±0.66	NT	NT	NT	NT	NT	NT
30 s	NT	NT	3.42±0.88*	NT	NT	4.75±0.43	NT	NT	NT	NT	NT	NT
1 min	NT	NT	NT	NT	NT	3.50±0.87*	NT	NT	NT	NT	NT	NT
5 min	NT	NT	NT	NT	NT	NT	6.25±0.90	5.33±0.63	5.58±0.29	6.83±0.80	NT	NT
10 min	NT	5.42±0.14	NT	NT	NT	NT	4.67±0.29*	3.92±1.01*	3.92±1.13*	6.08±0.38	5.42±0.80	5.42±0.38
30 min	6.67±0.29	4.00±0.50*	NT	NT	5.67±0.72	NT	3.25±0.75*	2.50±0.00*	2.58±0.14*	4.67±0.72*	3.33±0.72*	3.75±0.90*
1 h	6.75±0.25	NT	NT	NT	5.25±1.09	NT	NT	NT	NT	NT	NT	NT
2 h	6.92±0.29	NT	NT	6.92±0.25	3.67±0.63*	NT	NT	NT	NT	NT	NT	NT

a Fetal bovine serum was added to fresh charcoal ash and slaked lime solution as 5% organic materials of total volume.

b Concentration of fresh charcoal ash and slaked lime solution (%w/v).

c The titer converted into an index in log_10_ of virus control.

d Added 1M Tris-HCl before virus.

e The titer converted into an index in log_10_ of the recovered virus after indicated time of treatment such as 5 s, 30 s, 1 min, 5 min, 10 min, 30 min, 1 h, 2 h, 3 h, and 6 h.

f NT: Not tested.

* Inactivation effective when RF was ≥3

Table-3 shows NDV inactivation using both solution samples. In the absence of organic material of 5% and 10%, FCA solution could inactivate NDV within 2 h and 5 min, respectively. However, even in the presence of organic material of 10% FCA inactivated NDV within 10 min. Alongside, all concentrations of SL could inactivate NDV in the absence and presence of organic materials within 5 min and 10 min, respectively (Table-3).

**Table-3:** The results are shown as log_10_ TCID_50_/ml (mean±SE) of Newcastle disease virus inactivating activity by the mean of reaction in 1%, 5%, and 10% fresh charcoal ash and slaked lime solution in the absence or presence of organic materials.

Conditions	Fresh charcoal ash						Slaked lime					
	
	Absence of organic materials			Presence of organic materials^a^			Absence of organic materials			Presence of organic materials		
			
	1%^b^	5%	10%	1%	5%	10%	1%	5%	10%	1%	5%	10%
t_pc_^c^	8.08±0.38	8.17±0.29	8.08±0.38	8.08±0.38	8.08±0.38	8.08±0.38	8.00±1.09	8.00±1.09	8.00±1.09	8.00±1.09	8.00±1.09	8.00±1.09
0 s^d^	7.83±0.14	7.92±0.29	7.67±0.76	7.83±0.88	8.08±0.52	8.00±0.50	7.58±0.95	7.67±1.01	7.75±1.32	7.83±0.95	7.58±1.01	7.83±0.95
1 min	NT	NT	NT	NT	NT	NT	7.00±0.50	6.83±0.38	6.92±0.14	NT	NT	NT
3 min	NT	NT	5.25±0.66	NT	NT	6.25±0.43	5.08±0.38	4.75±0.25	4.92±0.38	5.92±0.76	5.92±0.72	5.58±0.76
5 min	NT	NT	4.67±0.29*	NT	NT	5.17±0.14	4.33±0.52*	4.17±0.63*	4.25±1.09*	5.33±0.76	4.42±1.04	4.92±0.58
10 min	NT	NT	4.33±0.29*	NT	NT	4.58±0.14*	NT	NT	NT	3.92±0.80*	4.00±0.90*	3.83±0.95*
30 min	7.17±0.52	6.50±1.39	NT	8.17±0.29	7.50±0.66	NT	NT	NT	NT	NT	NT	NT
1 h	7.25±0.90	5.08±0.58	NT	7.58±0.38	7.00±0.87	NT	NT	NT	NT	NT	NT	NT
2 h	7.17±0.95	3.38±1.15*	NT	7.33±0.72	6.92±0.76	NT	NT	NT	NT	NT	NT	NT

a Fetal bovine serum was added to fresh charcoal ash and slaked lime solution as 5% organic materials of total volume.

b Concentration of fresh charcoal ash and slaked lime solution (%w/v).

c The titer converted into an index in log_10_ of virus control.

d Added 1M Tris-HCl before virus. ^e^The titer converted into an index in log_10_ of the recovered virus after indicated time of treatment such as 5 sec, 30 sec, 1 min, 5 min, 10 min, 30 min, 1 h, 2 h, 3 h, and 6 h. ^f^NT=Not tested.

* Inactivation effective when RF was ≥3

Under the wet and dry conditions during a 3-min incubation period, FCA was demonstrated to effectively inactivate AIV or NDV when resuspended 1 and 2 times, respectively; however, SL was found effective when resuspended 12 and 9 times, respectively (Table-4). In addition, the stability of the sample solution is shown in Table-5. FCA solution still inactivates AIV and NDV after preparing and keeping it at room temperature for at least 8 weeks as 10% solution, while as SL could inactivate AIV at 10% solution at 4-week post preparing and at least 2 weeks for NDV inactivation at 10% (Table-5).

**Table-4:** The RF (log_10_ TCID_50_/ml) of fresh charcoal ash and slaked lime applied for inactivating Newcastle disease virus and Avian influenza virus under wet and dry conditions at consecutive re-suspension times with a 3-min incubation period.

Number of times resuspended	Avian influenza virus		Newcastle disease virus	
	
	Fresh charcoal ash	Slaked lime	Fresh charcoal ash	Slaked lime
1	>5.00*	>4.75*	>5.75*	>5.00*
2	0.50	>4.75*	5.25*	>5.50*
3	0.50	>4.75*	0.00	>5.50*
4	0.50	>4.75*	0.50	>5.50*
5	0.50	>4.75*	NT	>5.50*
6	NT	>5.00*	NT	>5.50*
7	NT	>5.00*	NT	>5.50*
8	NT	>5.00*	NT	3.25*
9	NT	>5.00*	NT	3.75*
10	NT	>5.00*	NT	2.50
11	NT	>5.00*	NT	1.00
12	NT	>4.75*	NT	0.00
13	NT	2.50	NT	NT
14	NT	2.25	NT	NT
15	NT	1.75	NT	NT

* Inactivation regarded effective when RF was ≥3, RF=Reduction factor

**Table-5:** The RF (log_10_ TCID_50_/ml) of fresh charcoal ash and slaked lime solution applied for inactivating Newcastle disease virus and Avian influenza virus after being kept at room temperature with a 3-min incubation period.

Time point (week)	Concentration (%)	Avian influenza virus		Newcastle disease virus	
	
		Fresh charcoal ash	Slaked lime	Fresh charcoal ash	Slaked lime
2	1	0.50	0.50	0.50	0.50
	5	0.50	2.50	1.75	0.50
	10	3.5*	3.75*	>5.50*	0.50
4	10	3.50*	3.25*	>4.25*	NT
6	10	3.25*	NT	>5.25*	NT
8	10	3.25*	NT	>5.0*	NT

* Inactivation regarded effective when RF was ≥3, RF=Reduction factor

## Discussion

The FCA or wood ash is the residue powder left after the combustion of wood, such as burning wood in a home fireplace or an industrial powder plant. The largest component of charcoal ash (about 25%) is CaCO_3_, <10% is potash, and <1% phosphate [5,6,11]. However, there are trace elements of iron, manganese, zinc, copper, and some heavy metals [11]. All of these are, primarily, in the form of oxides [12]. In addition, Demeyer *et al*. [5] and Tarun *et al*. [6] described the FCA as strong alkaline that contains main inorganic materials such as calcium oxide and Ca[OH]_2_. In water, calcium oxide changes to Ca[OH]_2_ [3], which has the ability by means of hydroxyl ion, to damage bacterial cytoplasmic membrane, cause protein denaturation by breakdown the ionic bonds, and damage DNA strand [13]. In the present study, it was shown that high pH of all FCA solutions could inactivate pathogens, mostly; however, when neutralized by Tris-HCl before treated, all solutions could not inactivate all pathogens. These results indicated that high pH underlies an important mechanism for inactivation, and Tris-HCl was used as an instrument for determination of exposure or contact time in the present study.

The SL is an inorganic compound, practically containing >70% Ca[OH]_2_. In general, Ca[OH]_2_ is a strong alkaline substance. Most of the enteropathogens are unable to survive in this high alkaline environment [14]. In general, SL is widely used as a disinfectant and involves problems associated with its use, such as corrosion and human irritation. The disinfection mechanism of SL is thought to act due to its high pH [15]. The present study showed virucidal ability of SL which affected to viruses in powder form and solution form.

In general, several disinfectants such as chlorine and quaternary ammonium compounds could not inactivate bacteria and viruses when contaminated with organic materials. However, the FCA and SL could inactivate AIV and NDV even in the presence of organic materials in the present study. These findings are compatible with results obtained by several researchers which tested alkaline agents such as food additive Ca[OH]_2_ (pH 12.5) and SL (pH12.5); that showed virucidal effect toward NDV [16]; calcinated eggshell powder (pH12.7), that showed virucidal ability against NDV, AIV and infectious bursal disease virus [4,17]; as well as scallop shell powder (pH 13.0) and SL that could inactivate AIV [3]. Lorcharoenrungroj *et al*. [18] reported that FCA could inactivate AIV, *Escherichia coli*, and *Salmonella* infantis in the presence of organic material and those findings are related to the present study. Finally, not only alkaline agent that could inactivate viruses even in the presence of organic material but also acidic agents were pointed at by Sonthipet *et al*. [8], who described the bactericidal and virucidal efficacies of potassium monopersulfate (pH 2.04); this acidic agent also inactivated AIV on virus-spiked clothes.

In addition, the wet-dry conditions and the stability of the solution sample were illustrated and lasted long enough to inactivate AIV and NDV under both conditions. The efficacy of wet-dry conditions of FCA is limited when compared with SL. This result indicated that the stability of FCA powder is not steady, especially in the rainy season, when FCA powder may be washed or soaked by rain, thereupon being affected similarly to the wet conditions in the present study. On the other hand, it was demonstrated that the FCA solution was more stable and kept at room temperature longer than SL. These stability results may be applied, suggesting an alternative disinfectant agent, especially for biosecurity enhancement on and around chicken farms.

## Conclusion

Both powder and solution forms of FCA and SL could inactivate AIV and NDV under various concentrations, organic material presence, and during exposure or contact timing. Thereby, FCA might be used as an alternative material, while applying it to prevent spreading of poultry disease on commercial chicken farms and also backyard chickens, especially in developing countries, including in rural areas of Thailand.

## Authors’ Contributions

KT supervised the present study. SR designed and coordinated the study. TJ, SR, DP and KT performed the experiment. SR analyzed the data and wrote the manuscript. The final manuscript has been read and developed in consultation with all authors.
